# Staphylococcal enterotoxin B suppresses Alix and compromises intestinal epithelial barrier functions

**DOI:** 10.1186/1423-0127-21-29

**Published:** 2014-04-09

**Authors:** Hao Yan, Haitao Yi, Lixin Xia, Zhenke Zhan, Weiyi He, Jijuan Cao, Ping-Chang Yang, Zhigang Liu

**Affiliations:** 1 The first affiliated hospital of Liaoning Medical college, Jinzhou, China; 2 Shenzhen Key Laboratory of Allergy & Immunology, Shenzhen University School of Medicine, Shenzhen, China; 3 Liaoning Entry-Exit Inspection and Quarantine Bureau, Dalian, China

**Keywords:** Alix, Epithelial cell, Barrier function, Staphylococcal enterotoxin B, Antigenicity

## Abstract

**Background:**

The epithelial barrier dysfunction plays a critical role in the pathogenesis of a broad array of immune diseases. Alix protein is involved in the endolysosome system. This study aims to elucidate the role of Alix in the maintenance of epithelial barrier function.

**Results:**

The results showed that Alix was detected in T84 cells at both mRNA and protein levels. Exposure to Staphylococcal enterotoxin B (SEB) markedly suppressed the expression of Alix in T84 cells, which could be blocked by knocking down the Toll like receptor 2. The exposure to SEB did not affect the TER, but markedly increased the permeability of T84 monolayers to OVA; the OVA passing through T84 monolayers still preserved the antigenicity manifesting inducing antigen specific T cells proliferation.

**Conclusions:**

Alix protein plays a critical role in the maintenance of the barrier function of T84 monolayers.

## Background

The epithelial barrier indicates the epithelial cell layer on the surface of mucosa such as airway and intestine. The epithelial barrier dysfunction is recognized in a number of body disorders, such as intestinal allergy [1], inflammatory bowel diseases [2] and asthma [3]. The pathogenesis is unclear. Our previous studies reveal that microbial products, such as Staphylococcal enterotoxin B (SEB), can facilitate the development of immune disorders in the intestine [4]. However, how the microbial products passing through the epithelial barrier to arrive the deep part of tissue is elusive.

The dysfunction of epithelial barrier manifests increases in the permeability to macromolecular molecules, such as protein antigens. The macromolecular substances may pass through the paracellular spaces, or to be transported via the transcellular pathway, to arrive the subepithelial region. Under healthy condition, epithelial cells endocytose some proteins of small molecular weight; those endocytic cargo can be wrapped by the plasma membranes to be formed as endosomes; the latter fuse with lysososmes where there are acidic enzymes to degrade the endocytic cargo. Recent reports indicate that there are a number of factors can affect the endolysosome systems to enhance the epithelial barrier permeability [5-7]; the causative factors include microbial products, such as cholera toxin [5] and SEB [8]. The underlying mechanism remains to be further understood.

Alix/Aip1 (Alix, in short) is a protein that functions in endosomal protein sorting, enveloped virus budding, and many other cellular processes. Crystal structures show that the Alix protein is composed of an N-terminal Bro1 domain and a central domain, the latter consists of two extended three-helix bundles that form elongated arms that fold back into a “V” [9]. Alix binds to the endosomal sorting complex required for transport (ESCRT) to facilitate the membrane fusion events during the multivesicular endosome formation [10]. Based on the above information, we hypothesize that Alix is involved in the transcellular transport in epithelial cells. In this study, we observed that intestinal epithelial cell line, T84 cell, expresses Alix. Exposure to SEB suppressed the expression of Alix in T84 cells, which resulted in enhancing the epithelial barrier permeability to macromolecular antigens.

## Methods

### Reagents

The antibodies of Alix (1A12), TLR2 (H-175), shRNA of TLR2 and shRNA of Alix were purchased from Santa Cruz Biotech (Shanghai, China). The reagents of qRT-PCR, Western blotting and gene cloning were purchased from Invitrogen (Shanghai, China). SEB was purchased from Sigma Aldrich (Shanghai, China). The immune cell isolation kits were purchased from Miltenyi Biotech (Shanghai, China). The OVA ELISA kit was purchased from Antibodies Online (Atlanta, GA).

### Mice

The OVA-TCR transgenic DO11.10 mice (8-10 week old) were purchased from the Xian Experimental Animal Center (Xian, China). The mice were maintained in a pathogen free environment.

### Cell culture

T84 cells were purchased from ATCC (American Type Culture Collection). Passages 33-38 were used in the study. The cells were cultured in DMEM (Dulbecco’s Modified Eagle Medium) supplemented with 10% fetal bovine serum, 2 mM L-glutamine, 100 U/ml penicillin and 0.1 mg/ml streptomycin. Cells were seeded onto the inserts of Transwells at 10^6^ cells/ml. The medium was changed daily.

### Recording transepithelial electric resistance (TER)

The TER was measured with an Ohmmeter following our established procedures [11].

### Assessment of T84 monolayer permeability

After the confluence (TER ≥ 1000 Ω. cm^2^) of the T84 monolayers, the OVA was added to the Transwell apical chambers at a concentration of 10 μg/ml. Samples were collected from the basal chambers 48 h later. The contents of OVA in the samples were determined by ELISA (Enzyme-linked immunosorbent assay) with a commercial reagent kit following the manufacturer’s instructions.

### Quantitative real time RT-PCR

Total RNA was extracted from T84 cells with the TRIzol reagents. The cDNA was synthesized with a reverse transcription kit. qPCR was performed in a real time PCR system (MiniOpticon, Bio-Rad, Shanghai, China) with the SYBR Green Super Mix. The results were calculated with the 2^-ΔΔCt^ method. The primers using in this study include: Alix, forward, aaggaacgttggcaaaggac; reverse, gaagggatggcagcattcag. β-actin, forward, cgcaaagacctgtatgccaa; reverse, cacacagagtacttgcgctc.

### Western blotting

Total proteins were extracted from T84 cells, fractioned by SDS-PAGE (sodium dodecyl sulfate polyacrylamide gel electrophoresis) and transferred onto a nitrocellulose membrane. The membrane was blocked by 1% bovine serum albumin (BSA) and incubated with the primary antibodies (0.5-1 μg/ml) for 1 h at room temperature, and followed by incubation with the secondary antibodies (conjugated with horseradish peroxidase) for 1 h. Washing with TBST (Tris-buffered saline-Tween 20) was performed after each incubation. The immune blots on the membrane were developed with ECL (enhanced chemiluminescence). The results were recorded with x ray films.

### RNA interference (RNAi)

**Figure 1:**
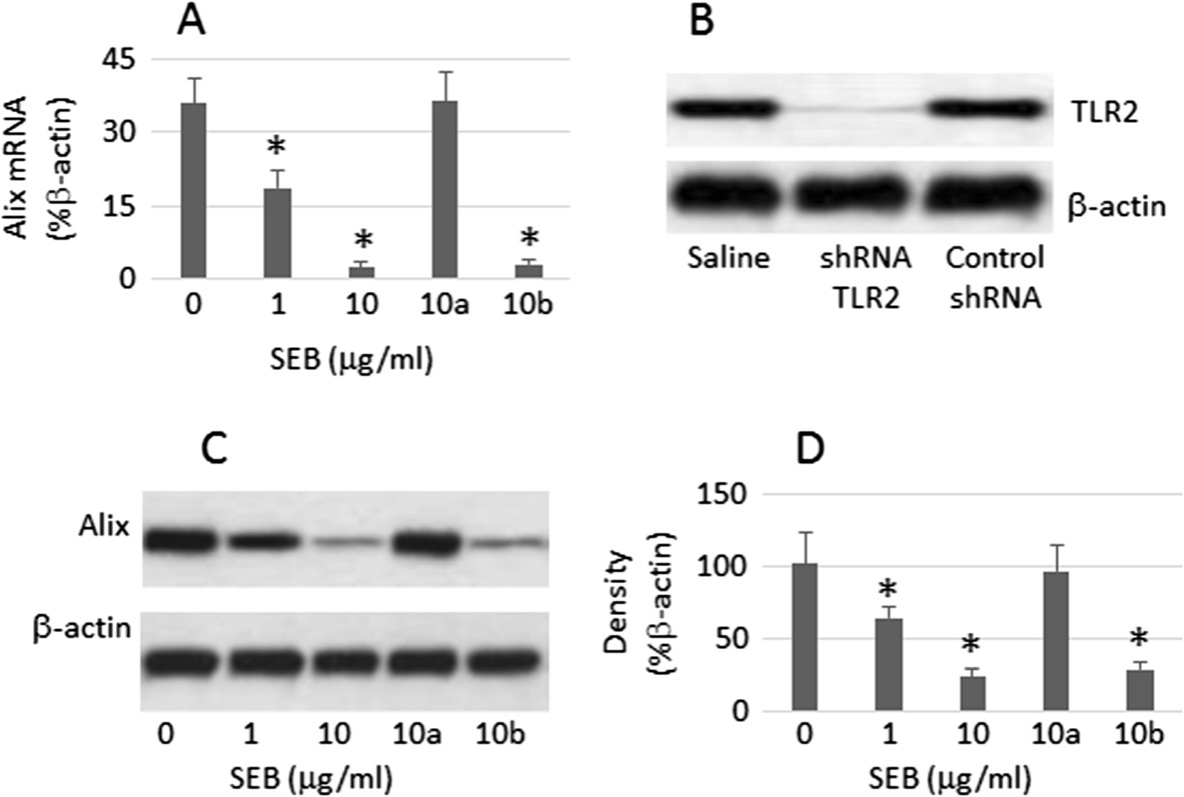
**Activation of TLR2 suppresses Alix in T84 cells.** T84 cells were stimulated with SEB in the culture for 48 h. **A**, the bars indicate the mRNA levels of Alix. **B**, the immune blots show the results of Alix RNAi. **C**, the immune blots indicate the protein contents of Alix. **D**, the bars indicate the summarized density of the immune blots of panel C. a, TLR2-null T84 cells; b, T84 cells were treated with control shRNA. The data of bars are presented as mean ± SD. *, p < 0.01, compared with dose “0” group. The data represent 3 separate experiments.

**Figure 2:**
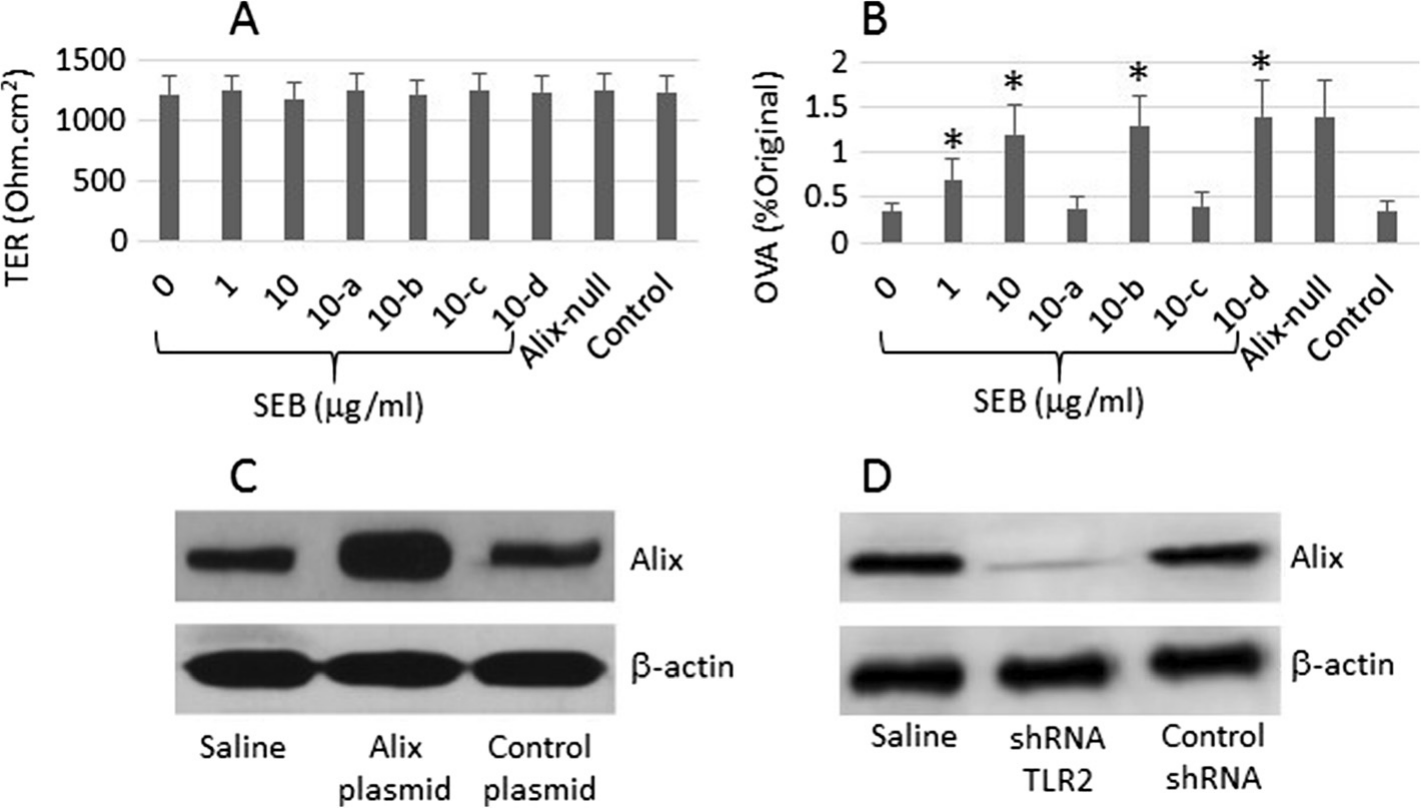
**SEB increases transcellular permeability of T84 monolayers.** T84 monolayers were stimulated with SEB (the doses were denoted on the x axis of **A** and **B**) in Transwells for 48 h. **A**, the bars indicate the TER of the T84 monolayers. **B**, the bars indicate the OVA levels in the Transwell basal chambers; the OVA was expressed as a percentage of the OVA amounts added to the Transwell apical chambers. **C**, the immune blots show the Alix over expression results. **D**, the immune blots indicate the Alix gene knockdown results. The data of bars are presented as mean ± SD. *, p < 0.01, compared with the SEB dose “0”. The data represent 3 separate experiments.

T84 cells were treated with RNAi to knock down the genes of Alix or Toll like receptor 2 (TLR2) with commercial reagent kits following the manufacturer’s instructions. The effect of gene knockdown was checked by Western blotting. The results of gene silence reached its peaked value on day 4 after the transduction and lasted at least 4 weeks. The data are presented in Figures 1 and 2 respectively).

### Over expression of the Alix gene

T84 cells were washed with phosphate buffered saline (PBS); the genomic DNA was extracted from T84 cells. The Alix gene was amplified by PCR. The products of PCR were sequenced first and confirmed, and cloned into the pTZ57R/T vector and transformed into E. coli. The vectors of Alix gene were subcloned into the pcDNA3 plasmid, and transformed into competent E. coli by the heat shock method. The plasmid was then purified using a plasmid extraction kit according to the manufacturer’s instructions. The presence of the Alix gene was confirmed by sequencing. T84 cells were transfected with the constructed plasmids using a transfection kit according to the manufacturer’s instructions. The over expression of Alix was assessed in Western blotting.

### Isolation of immune cells

CD4^+^ CD25^-^ effector T cells (Teff cell) and dendritic cells (DC) were isolated from DO11.10 mouse spleen with commercial reagent kits following the manufacturer’s instructions. The purity of isolated Teff cells was 98.8%, DC was 99.2% respectively as assessed by flow cytometry.

### Teff cell proliferation

The isolated Teff cells were labeled with CFSE (carboxyfluorescein diacetatesuccinimidyl ester), cultured with the supernatant collected from the Transwell basal chambers for 3 days in the presence of DC at a ratio of 1:5 (DC:T cell). The cells were analyzed by flow cytometry to determine the frequency of T cell proliferation.

### Statistics

The data are presented as mean ± SD. Differences between groups were determined by ANOVA. P < 0.05 was set as a significant criterion.

### Ethical approval

The animal experiments were approved by the Animal Ethic Committee at Shenzhen University.

## Results

### Exposure to SEB suppresses the expression of Alix in T84 monolayers

In the first attempt, we assessed the expression of Alix in T84 cells. The results of qRT-PCR and Western blotting showed that Alix was detected in T84 cells. Next, we stimulated T84 cells with SEB in the culture for 48 h; the cells were then collected and processed to assess the expression of Alix. The results showed that the levels of Alix were suppressed in T84 cells in a SEB dose-dependent manner. To elucidate the role of TLR2 in the SEB-induced suppression of Alix in T84 cells, in separate experiments, the TLR2 gene was knocked down in T84 cells by RNAi; the TLR2-null cells were exposed to SEB in the culture for 48 h. Indeed, the expression of Alix was not affected in T84 cells (Figure 1). The results indicate that T84 cells express Alix that can be suppressed by SEB through the TLR2 activation.

### Suppression of Alix compromises T84 monolayer permeability

Alix is associated with the endolysosome system in the cell. The endolysosome system is critical in the degradation of the endocytic cargo, such as protein antigens. To elucidate if Alix suppression plays any roles in the intestinal epithelial barrier permeability, we prepared T84 monolayers; the monolayers were treated with SEB with similar procedures of Figure 1. The TER and permeability to OVA of T84 monolayers was assessed. The results showed that the exposure to SEB did not affect the TER, but significantly increased the permeability to OVA, which was abolished by Knockdown of TLR. To corroborate the results, we knocked down the Alix gene of T84 cells. The Alix-null T84 cells still formed monolayers in Transwells with comparable TER with wild control T84 cells. Then, we assessed the permeability of the Alix-null T84 monolayers. The results showed that the Alix-null T84 monolayers had markedly higher permeability to OVA as compared with wild T84 monolayers (Figure 2). The results indicate that SEB can increase the permeability to OVA via suppressing Alix.

### Antigens passing through SEB-treated T84 monolayers preserve antigenicity

**Figure 3:**
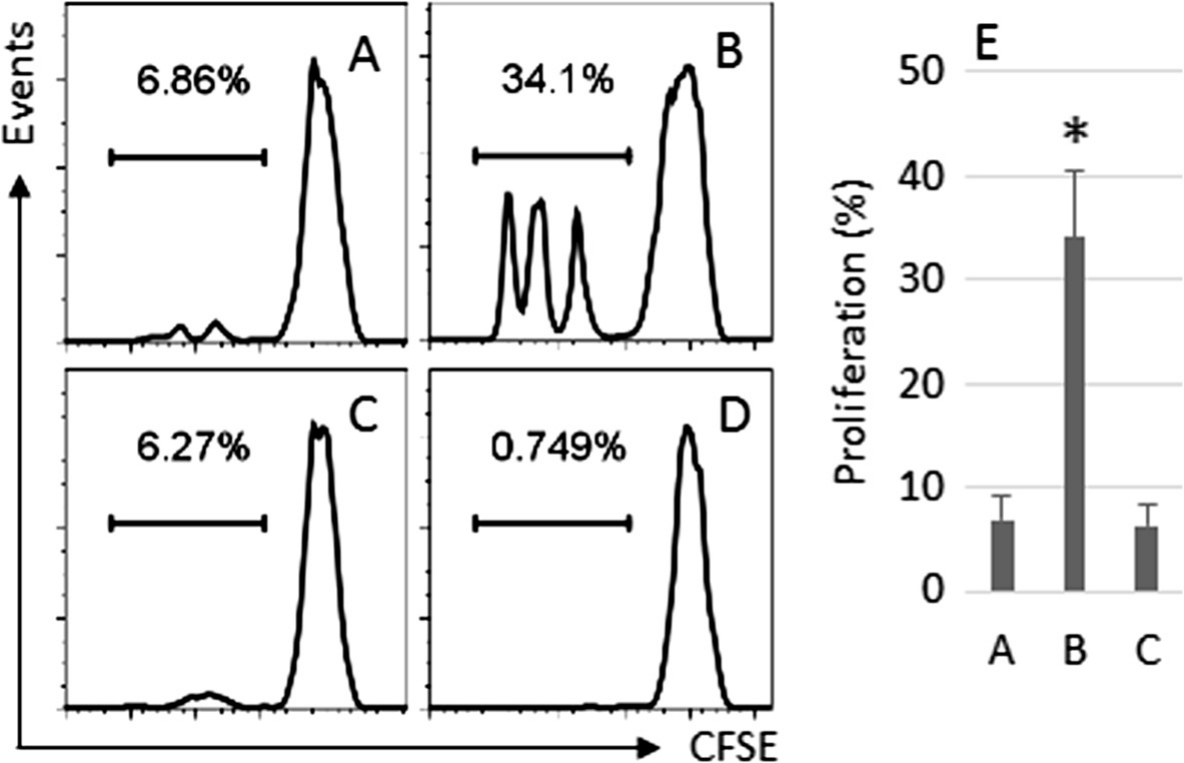
**OVA from Transwell basal chambers preserves antigenicity.** CD4^+^ CD25^-^ T effector (Teff) cells (labeled with CFSE) and DCs were isolated from the DO11.10 mouse spleen. The Teff cells and DCs were cultured at a ratio of 5:1 (T cell:DC) in the supernatant collected from the Transwell basal chambers. Apart from adding OVA to the apical chambers, the additional treatment included supernatant alone **(A)**, SEB **(B)**, Alix-over expressing T84 monolayers treated with SEB **(C)**. Three days later, the cells were analyzed by flow cytometry. **A-C**, the histograms indicate the Teff cell proliferation. **D** is a no staining control. **E**, the bars indicate the summarized data from the histograms, the labels of x axis are the same as those of histograms. The data of **E** are presented as mean ± SD. *, p < 0.01, compared with group **A**. The data represent 3 separate experiments.

The data of Figure 2 implicate that the OVA collected from the Transwell basal chambers still has antigenicity. To test the hypothesis, we isolated OVA-specific CD4^+^ CD25^-^ T effector (Teff) cells from DO11.10 mouse spleen, and cultured the cells with the supernatant collected from the Transwell basal chambers in which OVA might be transported from the apical cambers passing through the T84 monolayer. As shown by the data of flow cytometry, the proliferation frequency of the Teff cells was 6.86% in medium alone group, 34.1% in the SEB stimulated group and 6.27% in the group with Alix over expression and stimulated with SEB (Figure 3). The results indicate that the OVA passing through the T84 monolayers still preserves the antigenicity.

## Discussion

Epithelial barrier dysfunction is one of the major causative factors in the pathogenesis of a large number of immune diseases; the underlying mechanisms are not fully understood yet. The present study has revealed that intestinal epithelial cell line, T84 cells, expresses Alix. Exposure to a microbial product, SEB, markedly suppresses the expression of Alix in the epithelial cells, which results in the epithelial barrier dysfunction.

Although the precise mechanism remains obscure, cumulative reports indicate that multiple factors are involved in the induction of epithelial barrier dysfunction. Our previous studies indicate that high levels of IL-4 and atopic serum can significantly decrease T84 monolayer resistance and increased transepithelial horseradish peroxidase (HRP) transport. HRP transport induced by IL-4 can be inhibited by cold (4°C) environment and the tyrosine kinase inhibitor genistein [11]. Epithelial cells express CD23 on the surface that facilitates the transcellular transport of specific antigens across the epithelial barrier [12,13]. Recent reports indicate that exposure to microbial products also affects the epithelial barrier functions [5,8]. The present study adds novel information to this area by showing that Alix is required in the maintenance of the epithelial barrier function. Exposure to microbial product, SEB, can inhibit the expression of Alix in epithelial cells which contribute to the hyperpermeability of the epithelial barrier.

Alix can bind to ESCRT, plays a role in the endosome/lysosome fusion. Sadoul proposed that the normal function of Alix in the endolysosomal system may be deviated by ALG-2 (apoptosis-linked gene 2) towards a destructive role during active cell death. Our data have added a piece of novel information that Alix is required in the degradation of the endocytic proteins in epithelial cells. It is proposed that Alix acts as a putative effector involving in membrane invagination, vesicle formation and fusion of endosomes and lysosomes in the controlling intracellular membrane traffic [14]. Our data provide further supporting evidence that Alix is required in the degradation of the endocytic protein antigens in epithelial cells. The underlying mechanism needs to be further investigated.

We also tested the antigenicity of the antigens to be transported across the T84 monolayers. The results showed strong antigenicity of the OVA in the supernatant collected from the Transwell basal chambers. Our previous studies indicate that upon the epithelial barrier dysfunction, a large quantity of macromolecular antigens can be transported into the deep region of the intestinal mucosa. Consequently, an intestinal allergy may be induced [15,16]. It is suggested by previous studies that multiple factors are involved in the regulation of the degradation of the endocytic proteins in epithelial cells; such as ubiquitin editing enzyme A20 is required in the endosome/lysosome fusion, which can be disturbed by inhibition of A20 resulting in incompletely degradation of the endocytic antigens [6,8]. Inhibition of myosin by tumor necrosis factor also induces intestinal epithelial barrier dysfunction [17]. Our data have added one more factor to the knowledge pool of epithelial barrier studies by showing evidence that Alix is required in maintaining epithelial barrier function.

It is noteworthy that exposure to SEB in the culture does not affect the TER as shown by the present data. The results implicate that the paracellular pathway is not influenced by SEB. The results are in line with previous studies. Lu et al indicate that SEB can activate monocytes to release proinflammatory cytokines to increase epithelial barrier permeability, but exposure to SEB alone does not affect TER [18]; such an abnormality may be prevented by the addition of transforming growth factor-β2 [19]. Our data indicate that the over expression of Alix also has the inhibitory effect on SEB-induced epithelial barrier dysfunction. Previous studies suggest that SEB facilitates the development of intestinal allergy via modulating dendritic cell properties or act as an adjuvant [4,20]. The present data provide novel information that SEB also compromises the transcellular antigen transport in the epithelial barrier.

## Conclusions

The present data show that human intestinal epithelial cell line, T84 cells, expresses Alix, which can be inhibited by SEB to induce epithelial barrier dysfunction. Over expression of Alix has the potential to attenuate the abnormally high epithelial barrier permeability.

## Competing interest

The authors declare that they have no competing interests.

## Authors’ contribution

HY, HY, LX, ZZ, WH, JC and SH performed the experiments, analyzed data and reviewed the manuscript. ZL and PCY designed the project, supervised the experiments and wrote the paper. All authors read and approved the final manuscript.
